# Identification of a novel drug-resistant community-acquired *Nocardia* spp. in a patient with bronchiectasis

**DOI:** 10.1080/22221751.2022.2069514

**Published:** 2022-05-23

**Authors:** Zhengtu Li, Yongming Li, Shaoqiang Li, Zhun Li, Ying Mai, Jing Cheng, Danhong Su, Yangqing Zhan, Nanshan Zhong, Feng Ye

**Affiliations:** a State Key Laboratory of Respiratory Disease, National Clinical Research Center for Respiratory Disease, Guangzhou Institute of Respiratory Health, The First Affiliated Hospital of Guangzhou Medical University, National Center for Respiratory Medicine, Guangzhou, People’s Republic of China; b Clinical Microbiology Laboratory, The First Affiliated Hospital of Guangzhou Medical University, Guangzhou, People’s Republic of China

**Keywords:** *Nocardia*, antibiotic resistance, novel species, chronic lung diseases, bronchiectasis

## Abstract

A previously unknown *Nocardia* species was isolated from the lung tissue and bronchoalveolar lavage fluid (BALF) of a 58-year-old woman with bronchiectasis and recurrent pneumonia. This *Nocardia* (GZ2020^T^), which grew readily in Columbia blood agar and could induce pneumonia in a mouse model, represents a novel *Nocardia* species, and its closest known relatives are *Nocardia anaemiae* NBRC 100462^T^, *Nocardia pseudovaccinii* NBRC 100343^T^ and *Nocardia vinacea* NBRC 16497^T^. However, unlike all previously known species, GZ2020^T^ is the first genus of *Nocardia* spp. that is not susceptible to multiple drugs but does show susceptibility to linezolid and moxifloxacin, and thus, GZ2020^T^ potentially represents a substantial health threat to patients with bronchiectasis and immunocompromised individuals. Although the original pathogen source and method of spreading remain uncertain, a mode of transmission from the environment to humans could exist. Vigilance with respect to its spread in the population and the transmission of antibiotic resistance genes in the environment should be maintained.

## Introduction

*Nocardia* spp. are filamentous, aerobic, gram-positive, weakly acid-fast, bacilliform, branched bacteria that were first isolated by Edmond Nocard in 1888 from samples of a bovine farcy case [1]. Over the past several hundred years, *Nocardia* infections have been continuously reported worldwide [2]. Compared with other pathogens, these bacteria are common pathogens in immunosuppressed patients and can sometimes cause high mortality [3,4]. However, in recent years, an increasing number of reports have indicated that patients with chronic lung diseases (CLDs), such as chronic obstructive pulmonary disease (COPD) and bronchiectasis, can be affected [5,6]. Due to the increasing number of people at risk and the development of molecular diagnostic methods, a gradual increase in the incidence of *Nocardia* infection has been observed [5,7]. More than 100 species have been identified to date; many of them (over 30 strains) are thought to be associated with human diseases, and the majority of strains exhibit susceptibility to first-line antibiotic treatment [8]. Here, we report a confirmed case of a novel, highly drug-resistant community-acquired *Nocardia* infection as well as its diagnosis and treatment processes.

## Case report

**Figure 1.**
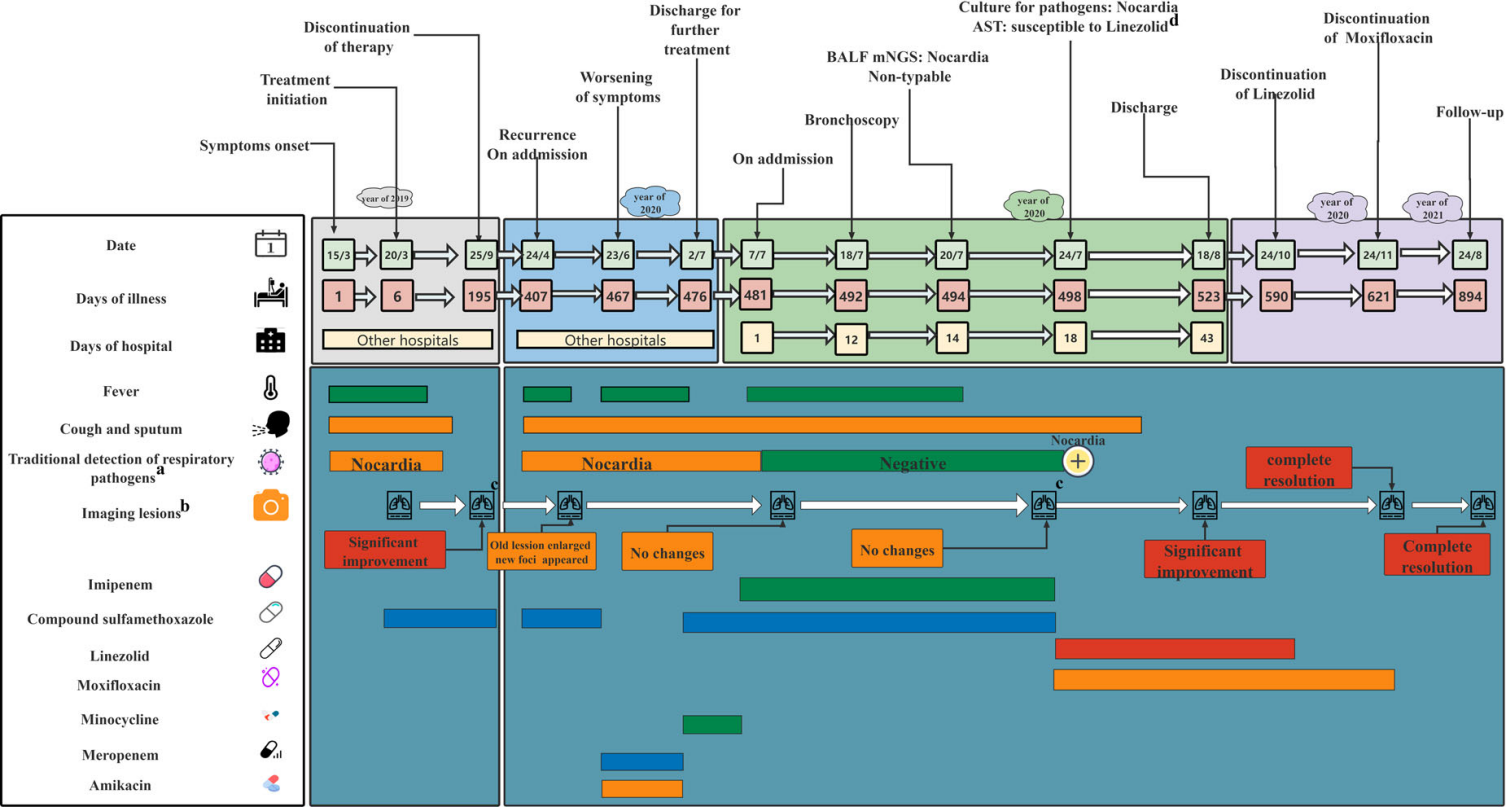
Timeline of the patient’s clinical course. Clinical course of the patient’s symptoms and treatment according to the day of illness and day of hospitalization from March 15, 2019, to August 24, 2021. BALF, bronchoalveolar lavage fluid; mNGS, metagenomic next-generation sequencing; AST, antimicrobial susceptibility testing. a: Detection of viruses, fungi, tuberculosis bacteria and other bacteria. Additional details are provided in Supplementary Table 6. b: The patient underwent CT examinations on March 20, 2019; September 20, 2019; May 27, 2020; July 8, 2020; July 21, 2020; August 17, 2020; December 21, 2020; and August 24, 2021. Each CT examination was compared with the previous examination to assess whether the lesions had improved. The CT examinations performed on September 20, 2019, and July 21, 2020, are not shown in this article, and the others can be found in Figure 2. c: Data are not shown. d: AST results showed that GZ2020T is susceptible to linezolid and possibly to moxifloxacin.

**Figure 2.**
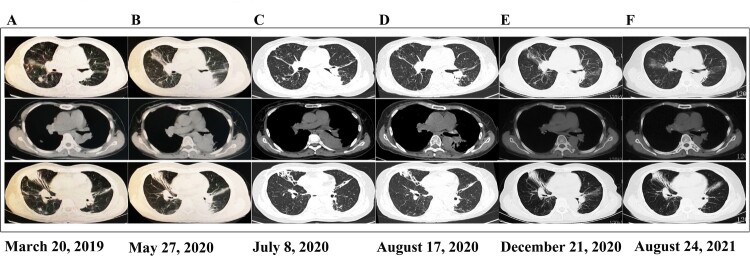
Imaging changes over time The upper section is the lung window, the middle section is the mediastinal window, and the lower section shows bronchiectasis lesions. A (March 20, 2019) shows the first chest CT obtained during the infection. Lesions were mainly observed in the upper lobe of the right lung, and bronchiectasis manifestation was observed, whereas in the lower lobe of the left lung, few significant lesions were seen. Subsequently, the patient was treated with trimethoprim-sulfamethoxazole (SMZ/TMP 400/80 mg; 0.96 g, po, q8 h) for 6 months. Her symptoms improved, and discontinuation of the medication was allowed. B (May 27, 2020) shows the first CT with recurrence of symptoms 1 year later—old lesions persisted in the right lung, and new-onset solid foci were observed in the left lung. She was then given SMZ/TMP (0.96 g, po, q8 h), meropenem (0.5 g, iv, q8 h) plus amikacin (0.4 g, iv, qd) and minocycline (100 mg, po, q12 h) plus SMZ/TMP (0.96 g, po, q8 h), but her symptoms were not completely resolved. C (July 8, 2020) shows the first chest CT scan obtained in our hospital, which showed no change compared with the last image. D (August 17, 2020) shows a chest CT scan after 24 days of linezolid (0.6 g, iv, q12 h) plus moxifloxacin (0.4 g, po, qd) treatment, which showed significant lesion absorption compared with the last image. E (December 21, 2020) shows a follow-up chest CT scan obtained after 4 months of linezolid (0.6 g, po, q12 h) plus moxifloxacin (0.4 g, po, qd) treatment and another 1 month of moxifloxacin monotherapy (0.4 g, qd, po), which resulted in complete absorption of the lesion. F (August 24, 2021) shows a follow-up chest CT scan obtained after drug withdrawal for more than 8 months, which showed no change compared with the last image. A, B, C, D, E and F indicate that precise antibiotic strategies were effective and necessary for treating the drug-resistant pathogen infection, and no exacerbation of bronchiectasis can be seen in these CT images.

In March 2019, a 58-year-old woman presented with fever, cough, and expectoration. She was diagnosed with pneumonia with *Nocardia* infection (*Nocardia* spp.; without antimicrobial susceptibility testing (AST)) at a local hospital (Figures 1 and 2A). Subsequently, trimethoprim-sulfamethoxazole (SMZ/TMP 400/80 mg; 0.96 g, po, q8 h) was administered empirically for six months and discontinued upon symptom improvement and partial absorption of lung lesions. Unfortunately, the above mentioned symptoms reoccurred and deteriorated in April 2020. A computed tomography (CT) scan of the chest revealed old expanded lesions, new-onset solid foci in the left lung, and bronchiectasis in both lungs (Figure 2B). A *Nocardia* spp. was identified as the primary pathogen in BALF culture, and SMZ/TMP (0.96 g, po, q8 h) was empirically administered again. The patient’s symptoms showed slight improvements after two months of treatment: the fever resolved, but the cough and expectoration were not completely eliminated. She was then discharged with continued outpatient antibiotic therapy. However, 4 days after discharge (June 20, 2020), the previous symptoms recurred again. A chest CT scan indicated that the lesions were similar to those imaged previously, and *Nocardia* spp. growth was observed in a sputum culture. Subsequently, the patient received combination therapy with meropenem (0.5 g, iv, q8 h) plus amikacin (0.4 g, iv, qd) from June 23 to July 1 and minocycline (100 mg, po, q12 h) plus SMZ/TMP (0.96 g, po, q8 h) from July 2–7, but the symptoms were not completely resolved. On July 7, 2020, she was transferred to our hospital for further treatment (Figure 1).

She had a history of bile reflux gastritis (in April 2019) but had been cured. She had no history of other underlying diseases, smoking or allergies. Physical examination revealed the following parameters: temperature, 39.5 °C; blood pressure, 95/56 mmHg; pulse, 132/min; respiratory rate, 20/min; and oxygen saturation, 98% while breathing ambient air. Laboratory tests (Supplementary Table 5) revealed a high white blood cell count of 17000 (normal range: 4000–10000) cells per cubic millimeter and a high neutrophil ratio of 85.9% (normal range: 40%−70%). The serum level of procalcitonin (PCT) was elevated at 0.2 ng/mL, and the erythrocyte sedimentation rate (ESR) was increased to 64 mm/h. However, pathogenetic examinations for viruses, fungi, Mycobacterium tuberculosis, and other bacteria (Supplementary Table 6) did not indicate the presence of any pathogen in sputum and blood specimens. Chest CT scans revealed bronchiectasis in both lungs and a large area of consolidation in the left lower lung (Figure 2C). Based on available evidence, the possibility of infection was high. Given the patient’s previous history of *Nocardia* infection and antibiotic treatment, imipenem and cilastatin sodium (1 g, iv, q6 h) plus SMZ/TMP (1.44 g, po, q8 h) were administered.

**Figure 3.**
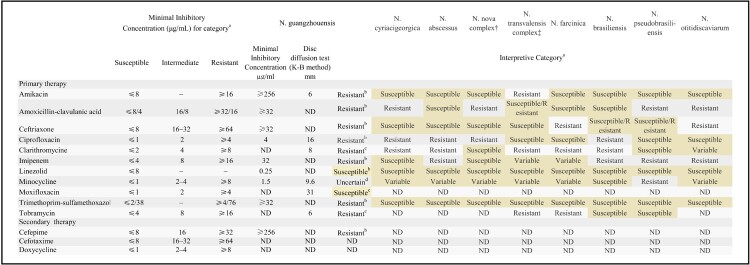
Drug resistance profile of GZ2020^T^. a: The data were obtained from reference 8 (Clinical and Laboratory Standards Institute (CLSI), M24-A2, ISBN 1-56238-746-4). b: Breakpoints defined by the CLSI for *Nocardia* spp. were applied. c: The results for tobramycin, clarithromycin and moxifloxacin were interpreted according to disc diffusion tests with the CLSI breakpoints for *Staphylococcus* spp. as a reference, which showed that GZ2020^T^ was resistant to tobramycin and clarithromycin and possibly susceptible to moxifloxacin. d: Although the result should be interpreted as “intermediate” according to the minimal inhibitory concentration (MIC) method, the poor clinical efficiency in the course of treatment and the markedly unsatisfactory outcomes from the disc diffusion tests instilled uncertainty. K-B method: Kirby-Bauer method; ND: no data.

Due to recurrent fever over the disease course, several malignancies and autoimmune diseases were ruled out before arriving at a definitive diagnosis. The patients tested negative for all autoimmune antibodies, but the levels of lung cancer markers were high (NSE: 21.48 ng/mL, CA125: 37.23 U/mL). Bronchoscopy was performed on July 18, which revealed bronchiectasis manifestations, and BALF and lung tissue were collected for pathogen detection. Subsequently, a metagenomic next-generation sequencing (mNGS) of BALF on July 20 showed *Nocardia* as the only pathogen (total reads: 50876 with *Nocardia* genus (49850 reads) accounting for the majority), but the species was unknown (Supplementary Table 7). Furthermore, the lung tissue and BALF culture results obtained on July 24 suggested the presence of *Nocardia* spp. (Supplementary Table 6). The histopathologic analysis results indicated inflammatory lung lesions. However, despite treatment from the time of admission (July 7) to July 23, the symptoms persisted. A repeat chest CT scan showed no obvious change in the lung lesion. Finally, the AST results on July 24 indicated that the pathogen was definitely susceptible to linezolid and possibly susceptible to moxifloxacin (Figure 3 and Supplementary Figures 4 and 5); subsequently, a combination of linezolid (0.6 g, iv, q12 h) plus moxifloxacin (0.4 g, po, qd) was administered. During 24 days of treatment, the patient’s symptoms showed gradual improvement, and a repeat chest CT scan revealed that the lesions were significantly absorbed on August 17 (Figure 2D). The patient was in satisfactory condition and discharged (August 18, 2020). As an anti-infection strategy, linezolid (0.6 g, po, q12 h) plus moxifloxacin (0.4 g, po, qd) was administered for another 3 months, moxifloxacin monotherapy (0.4 g, qd, po) was administered for more than 1 month, and the patient then opted to discontinue treatment for financial reasons. A follow-up chest CT scan (December 21, 2020, Figure 2E) revealed that the lesions had completely resolved, and this improvement in the clinical and microbial conditions has persisted steadily to date (August 24, 2021, Figure 2F), without any recurrence of pneumonia or exacerbation of bronchiectasis.

**Figure 4.**
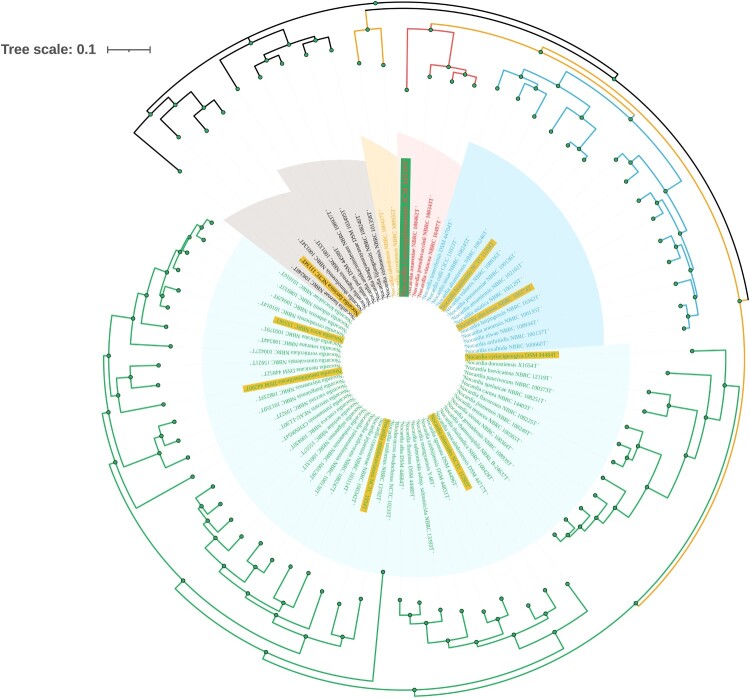
Phylogenetic tree analyses of GZ2020^T^. Species with a yellow background represent common *Nocardia* spp., and the strain with a green background represents GZ2020^T^. Different colored branches represent different groups.

**Figure 5.**
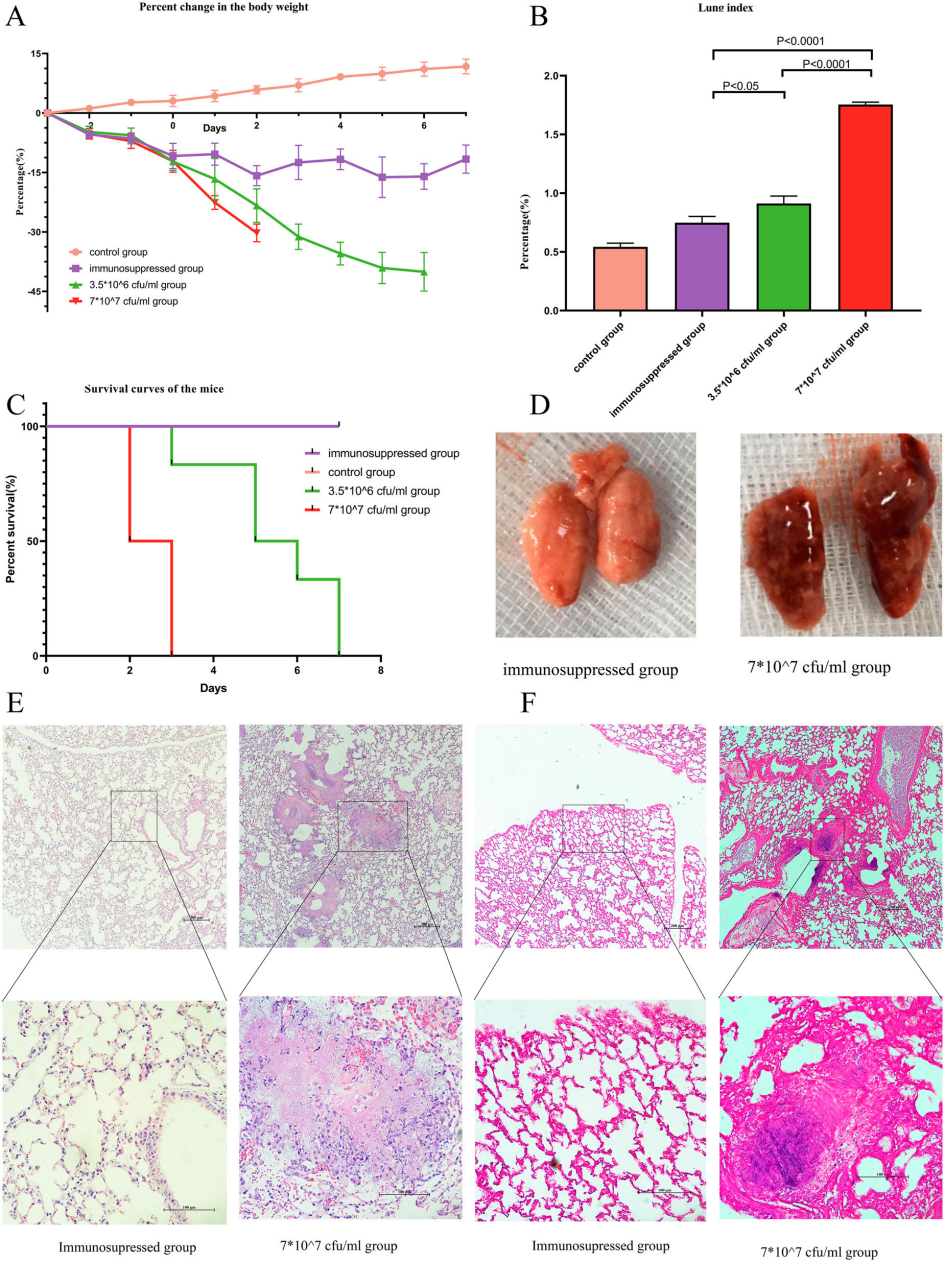
In vivo study of the bacterial pathogenicity. A: Percent change in body weight. B: Lung/body weight index of the groups. C Survival curves of the mice. Compared with the control and immunosuppressed groups, all mice in the infected groups died within 7 days, and the high-dose group (7*10^7/mL) showed 100% mortality at 72 h. D Gross pathology findings from the groups. Compared with the lungs of the control group, the lungs of the infected group were significantly congested and edematous. E (upper part: 100X magnification, lower part: 400X magnification) HE staining of samples from the immunosuppressed and infected groups (7*107 cfu/mL). Suppurative inflammatory changes, such as exudation, necrosis and infiltration of neutrophils, were observed in the infected group. F (upper part: 100X magnification, lower part: 400X magnification) Gram staining of samples from the immunosuppressed group (7*107 cfu/mL). Gram staining indicated rod-shaped bacterial aggregates in the lung of the infected group.

## Methods

### Specimen collection and bacterial culture

Blood, sputum, BALF and lung tissue samples were collected from the patient according to clinical practices [9,10]. In the laboratory, small aliquots of the samples were removed, plated onto Columbia blood agar (CBA) and incubated aerobically at 37 °C for 3 days. The bacteria were then prepared for staining and microscopic examination, including Gram staining, weak acid-fast staining, acid-fast staining and transmission electron microscopy.

## Genome sequencing and phylogenetic analysis

DNA was extracted from strain GZ2020^T^ using a nucleic acid extraction kit (Hangzhou Matridx Biotechnology). A 500-bp insert-size library was constructed with 0.2 μg of genomic DNA, and 136,235,859 single-end 75-bp clean reads were obtained using the NextSeq 500 platform. The sequencing data were assembled using Spades (v3.13.0), which resulted in 138 scaffolds of more than 1000 bp. The final genome sequence was annotated with Prokka (v1.14.6) and assessed with BUSCO (v4.1.4). Seventy-six previously published genome sequences of model *Nocardia* strains were obtained for phylogenetic tree construction, and the genomes and associated accession numbers are shown in Supplementary Table 1. For improved consistency, *Rhodococcus rhodochrous* NCTC 10210^T^ (LT906450), which is in the same family as *Nocardia*, was selected as the outgroup. The single nucleotide polymorphisms (SNP) information for 76 *Nocardia* strains was obtained using MUMmer (version 3.1) software [11,12]. Subsequently, to elucidate the exact taxonomic position of these novel *Nocardia* spp., a maximum-likelihood phylogenetic tree based on SNPs was constructed using FastTree (version 2.1.10) software [13]. The average nucleotide identity (ANI) [14] between the genomes was calculated using the Python module pyani based on MUMmer (ANIm) algorithms, and digital DNA:DNA hybridization (dDDH) similarities were computed with the Genome-to-Genome Distance Calculator (version 3, https://ggdc.dsmz.de/ggdc.php#). Subsequently, basic functional gene analysis was performed to compare with the other 3 relatives using eggnog (http://eggnog5.embl.de/)

## Physiological and chemotaxonomic analysis

All culture characteristics of strain GZ2020^T^ were determined by growing the strain on CBA at 37 °C for 3 days. Gram staining was conducted with Hucker’s method [15]. The morphological characteristics of the strain after growth on CBA at 37 °C for 3 days were observed by light microscopy (BX43, Olympus) and scanning electron microscopy (S-3000N, Hitachi). The growth of GZ2020^T^ on CBA was tested over 3 days of incubation at different temperatures (4, 10, 20, 28, 30, 37, 40 and 45 °C), with different NaCl concentrations (0–15%, w/v, at intervals of 1%) and at different pH values (4.0–12.0, at intervals of 1.0 pH units). Catalase activity was evaluated by observing whether bubbles were produced after pouring 3% H_2_O_2_ (v/v) on the colonies. The hydrolysis of starch and Tween (20, 80) was tested as described previously [16]. The utilization of single carbon sources was determined with a GEN III MicroPlate (Biolog) according to the manufacturers’ instructions. In vitro AST was performed using the E-test and disc diffusion test, as described previously for *Nocardia* species [17–19]. Breakpoints defined by the CLSI were applied for the E-test, [20,21] and the results from disc diffusion tests were interpreted according to the CLSI breakpoints for *Staphylococcus* spp.[18,22] The interpretation of antimicrobial susceptibility was based on the result of an E-test, and the disc diffusion test was used as a supplement when the E-test was unavailable. For further chemotaxonomic analyses, GZ2020^T^ was incubated in CBA for 3 days under aerobic conditions at 37 °C. The cells were harvested by centrifugation, washed twice with distilled water, recentrifuged and freeze-dried. The freeze-dried cell biomass was used to analyze polar lipids, quinones and cell sugars at the Guangdong Institute of Microbiology (Guangzhou, Guangdong, PR China), as described by Wu et al. [23] and Zhuang et al. [24].

## Bacterial pathogenicity in vivo

Forty-eight BALB/C mice were divided into 4 groups: high-dose infection group (7.0 x10^7^ cfu/mL, n = 12), low-dose infection group (3.5 x10^6^ cfu/mL, n = 12), immunosuppressed group (n = 12), and control group (n = 12). The mice in the infection and immunosuppressed groups were intraperitoneally injected with 150 mg/kg cyclophosphamide on days −3, −1, and +1 relative to infection, as in another study [24]. The experimental mice in the infection group were administered 50 µL of bacterial culture solution via nasal drip on day 0, and 50 µL of phosphate-buffered saline was administered to the mice in the immunosuppressed and control groups. On days 2 and 3, the mice were euthanized for lung pathologic examination assays and bacterial culture of lung tissue homogenates.

## Results

Under gross observation, colonies cultured on CBA plates were light yellow and circular and measured 0.5–1.0 mm in diameter after 3 days at 37 °C (Supplementary Figure 1). Bacteriologic analysis revealed positive Gram staining and weak acid-fast staining, and negative acid-fast staining was also observed (Supplementary Figure 2). Analysis of the cell morphology of this bacterial strain under an optical microscope revealed that the strain was beaded and had a rod shape with branching filaments. Scanning electron microscopy (SEM) analysis revealed a filamentous network covered with an opaque biofilm matrix (Supplementary Figure 3).

The genome (CDS) of GZ2020^T^ was found to have a length of 8,350,551 bp, with an average GC content of 66.89% (Supplementary Figure 6), which is similar to the genomes of other *Nocardia* spp. A maximum-likelihood phylogenetic tree based on SNPs was constructed using FastTree (version 2.1.10) software, and the results revealed that the closest known relatives were *Nocardia anaemiae* NBRC 100462^T^, *Nocardia pseudovaccinii* NBRC 100343^T^, and *Nocardia vinacea* NBRC 16497^T^, which form a distinct branch. However, considering the branch lengths, substantial genetic variation was observed between GZ2020^T^ and these 3 strains. Moreover, common species of *Nocardia* were even more distinct from GZ2020^T^ because these pathogens formed different branches in the phylogenetic tree, which belonged to different generations (Figure 4; details on the genetic, physiological and chemical characteristics are provided in the Supplementary Material).

The heatmap from the identity alignment of GZ2020^T^ with the other 3 pathogens showed ANI values < 95% and a similar dDDH result of less than 70% (Supplementary Figure 7). Physiological, chemotaxonomic, molecular biological methods and basic functional gene analysis indicated that this strain was somewhat different from known species (Supplementary Tables 2–4 and Supplementary Figure 8). Due to the gene analysis (ANI,dDDH and basic functional gene analysis) results and differences in physiological characteristics between GZ2020^T^ and these species, we concluded that GZ2020^T^ represented a novel *Nocardia* species and named it *Nocardia guangzhouensis* GZ2020^T^ ( = GDMCC 4.187^T ^= JCM 34519^T^). To further estimate its pathogenicity, in vivo virulence testing was performed.

Compared with the immunosuppressed and control groups, all infected mice died within 7 days, and the high dose group (7*10^7 cfu/mL) showed 100% mortality at 72 h (Figure 5C). Their body weight decreased progressively (Figure 5A), and the lung index increased significantly (Figure 5B). The lungs of the infected group were significantly congested and edematous (Figure 5D), and HE staining revealed suppurative inflammatory changes, such as exudation, necrosis and infiltration of neutrophils (Figure 5E). Gram staining indicated that rod-shaped bacteria aggregated in the lung (Figure 5F). Lung homogenates from the infected group were plated on CBA plates and cultured for gene sequencing, which showed that species isolated from the mice and GZ2020^T^ belonged to the same genus (Supplementary Figure 9).

## Discussion

Herein, we report a confirmed case of recurrent pulmonary infection caused by a novel community-acquired *Nocardia* species in Guangzhou, China. The patient did not belong to the traditional immunosuppressed population but had bronchiectasis. This pathogen is characterized by its low susceptibility to multiple antibiotics, including routine therapeutic antibacterial agents, SMZ/TMP, amikacin and imipenem. In vivo experiments indicated that the main injury mechanism was peribronchial purulent inflammation with massive neutrophilic infiltration. The substantial potential health threat posed by GZ2020^T^ deserves attention.

Bacteriological analysis revealed that GZ2020^T^ exhibited positive Gram staining and weak acid-fast staining, and microscopic examination showed that the bacteria tended to be beaded and to have a rod shape with branching filaments (Supplementary Figure 2), which is consistent with the common characteristics of *Nocardia* [1,2,6,25]. However, further phylogenetic analysis and physiological examinations (Figure 4 and Supplementary Tables 1–4) revealed that it belonged to the *Nocardia* species but was different from known strains, which indicated that GZ2020^T^ is a novel pathogen. Notably, compared with other *Nocardia* spp., GZ2020^T^ was isolated and propagated relatively easily on CBA plates, as demonstrated by the finding that bacterial colonies were visible to the naked eye within 72 h (Supplementary Figure 1 and 2A), which is the minimum time (48 h-72 h) needed before colonies of other *Nocardia* spp. are evident[25], and colonies of some for *Nocardia* spp. sometimes need 2–14 days to appear[26]. Most AR mechanisms are associated with a fitness cost, which typically manifests as a reduced bacterial growth rate in the absence of antibiotic pressure [27]. Hence, the growth rate of GZ2020^T^ indicates that this strain could be a naturally selective pathogen rather than a strain that evolved from a known species through antibiotic induction within a short time. However, the original pathogen source and method of spreading remain uncertain, and additional research is needed. Based on its gross appearance (Supplementary Figure 1), GZ2020^T^ appeared drier and rougher than known species, which may also contribute to its low susceptibility to multiple antibiotics, as previous studies have revealed [28,29].

Immunosuppressed patients were previously the main human population susceptible to *Nocardia* infection [2], but a history of bronchiectasis was also recently found to be a key risk factor [5,7,30]. It has been reported that the proportion of patients with bronchiectasis and *Nocardia* infection significantly increased from 11% during 1996–2001 to 33% during 2008-2013, and the increasing number of *Nocardia* infections over time could be driven by the occurrence of bronchiectasis instead of the immunocompromised status of the population [30]. Bronchiectasis is a predisposing condition for *Nocardia* infection, although the definite mechanism remains uncertain [7,30]. In addition, other CLDs, such as COPD, have been found to be related [4,5,7]; thus, attention should be appropriately shifted from the immunosuppressed population toward patients with CLD, particularly those with bronchiectasis. Changes in susceptible populations could be implicated in the alteration of the pathogenic spectrum and the emergence of novel strains. Enhanced surveillance is necessary for this population.

The most concerning health issue associated with GZ2020^T^ is its low susceptibility to first-line antibiotic treatment. The emergence and rapid spread of AR poses a severe threat to human health, and in 2019, AR could have been the third leading Global Burden of Diseases (GBD) Level 3 cause of death and was second only to ischemic heart disease and stroke [31]. The scenario might be even worse by 2050 if no effective measures are taken [32]. The majority of known *Nocardia* species are susceptible to first-line antibiotics, despite the emergence of some drug-resistant species[2,8]. However, GZ2020^T^ is the first *Nocardia* species to exhibit little susceptibility to multiple antibiotics (Figure 3), and the notable side effects of the most effective drug (linezolid) make its treatment challenging [33]. Although the original pathogen source and method of spreading remain uncertain, our animal model and this case demonstrate the existence of a possible mode of transmission from the environment to humans. Because this species was found in the community [34,35], assessment of the drug resistance burden caused by GZ2020^T^ could be difficult and complex. The current key to antibiotic resistance (AR) management lies in controlling the spread of resistant strains and genes [36]. Thus, much vigilance should be maintained for this novel pathogen and its spread. We will also perform detailed epidemiological research and analyses of resistance genes, which may be beneficial for understanding the development of drug resistance.

Community-acquired GZ2020^T^ could make the management of *Nocardia* more challenging in patients with bronchiectasis. Drug-resistant bacterial infections pose an increased risk of pathogen persistence and clinical deterioration [37]. Poorly controlled or recurrent chronic infections in patients with bronchiectasis could contribute to worsening of the quality of life, strengthening of bronchiectasis, deterioration of lung function [38], and increased mortality [39,40]. Therefore, a precise anti-infective strategy is necessary to rapidly eliminate pathogens and prevent the exacerbation of bronchiectasis. In this case, initial empiric antibiotic treatment was not sufficient to obtain a favorable effect. Subsequently, the administration of treatment guided by AST results gradually improved the symptoms and imaging results, and no clinical relapse or exacerbation of bronchiectasis has been observed to date (Figure 1), which indicates that the precise antibiotic strategies based on AST results were effective and necessary. Similarly, this pathogen could also increase the risk of immunocompromised patients. In addition, more than half of these *Nocardia* pneumonia patients could show a combination of extrapulmonary disease, and the brain is the most common site of metastasis [37]. When this pathogen breaks the blood–brain barrier, high mortality could be observed (even reaching 90% in cases of delayed treatment) [37]. Therefore, the emergence of GZ2020^T^ is of concern because this novel, multidrug-resistant pathogen could easily lead to late diagnosis and treatment and thus causes high mortality if central nervous system infections occur.

In conclusion, this case highlights the substantial health threat posed by novel, highly drug-resistant *Nocardia* spp. to patients with CLD and immunocompromised individuals, particularly those with bronchiectasis. Vigilance of its spread in the population and the transmission of AR genes in the environment should be maintained. This report also serves as a reminder that despite the achievements made to date, much more effort should be paid to the issue of AR.

## Declaration

The findings and conclusions in this report are those of the authors and do not necessarily represent the official position of the Centers for Disease Control and Prevention (CDC). The authors report no conflicts of interest. The authors alone are responsible for the content and writing of the paper. Informed consent and authorization were obtained from the patient, and the file is attached as Supplementary Material (Additional documents 1 and 2). All experiments involving *Nocardia* and other microorganisms in this article were carried out under standard biosecurity and institutional safety standards. All experiments were conducted in biosafety level 2 laboratories and strictly performed in accordance with laboratory biosafety regulations (procedurehttp://www.nhc.gov.cn/wjw/gfxwj/201304/64601962954745c1929e814462d0746c.shtml). Furthermore, this study was approved by the Ethics Committee of the First Affiliated Hospital of Guangzhou Medical University (ethical approval number: 2019-26).

GZ2020^T^ was identified and is preserved in the Guangdong Microbial Culture Collection Center (https://www.gdmcc.net/main.do?method=load&css=1&englist=) (GDMCC 4.187^T^) and Microbe Division, RIKEN BioResource Research Center, JAPAN COLLECTION OF MICROORGANISMS (https://jcm.brc.riken.jp/en/) (JCM 34519^T^) (Additional documents 3 and 4).

The raw sequence data for GZ2020^T^ identified in this study have been deposited under accession number GWHBEDI00000000 in the Genome Warehouse at the National Genomics Data Center, Beijing Institute of Genomics, Chinese Academy of Sciences/China National Center for Bioinformation, which is publicly accessible at https://ngdc.cncb.ac.cn/gwh. All other data are available from the authors upon reasonable request. Genome Warehouse: A Public Repository Housing Genome-scale Data. Genomics Proteomics Bioinformatics 2021. https://doi.org/10.1016/j.gpb.2021. 04.001; and Database Resources of the National Genomics Data Center, China National Center for Bioinformation in 2021. Nucleic Acids Res 2021, 49(D1): D18–D28.

The raw sequence data for GZ2020^T^ identified in this study have also been deposited in the NCBI database (accession number JAIRBR000000000).

The type strain, *Nocardia guangzhouensis* GZ2020^T^ ( = GDMCC 4.187^T ^= JCM 34519^T^), was isolated from the lung tissue and BALF specimens of a patient at the First Affiliated Hospital of Guangzhou Medical University in Guangzhou, Guangdong, China.

## Author contributions

Article design and writing: Zhengtu Li, Yongming Li, Shaoqiang Li, Nanshan Zhong, Feng Ye; patient management and treatment: Zhengtu Li, Shaoqiang Li, Yangqing Zhan, Nanshan Zhong, Feng Ye; clinical sample collection and detection: Ying Mai, Yongming Li, Zhun Li, Jing Cheng, Danhong Su. In vivo experiment: Yongming Li, Zhengtu Li, and Zhun Li.

All authors contributed to the acquisition, analysis, or interpretation of the data and reviewed and approved the final version of the manuscript.

## Supplementary Material

Supplemental Material
