# Supplementary material for: Identification of a novel drug-resistant community-acquired Nocardia spp. in a patient with bronchiectasis
Source: Emerg Microbes Infect. 2022 May 23;11(1):1346–55. doi: 10.1080/22221751.2022.2069514 (PMC9132467; doi:10.1080/22221751.2022.2069514)
Supplement: Supplemental Material [file TEMI_A_2069514_SM4929.pdf]

Supplementary Material

Contents

Supplementary Methods
mNGS analysis of BALF ……………………………………………………………..3

Supplementary Results and Discussion
Genomic features of GZ2020^T…………………………………………………......4

Physiological and chemical characteristics of GZ2020^T…………………………....5

Description of GZ2020^T…………………………………………………........6

Supplementary References…………………………………………….................7

Supplementary Tables
Table 1………………………………………………………………………8

Table 2……………………………………………………………………..10

Table 3……………………………………………………………………..11

Table 4……………………………………………………………………..13

Table 5……………………………………………………………………..15

Table 6……………………………………………………………………..17

Table 7……………………………………………………………………..19

Supplementary Figures
Figure 1……………………………………………………………………21

Figure 2……………………………………………………………………22

Figure 3……………………………………………………………………23

Figure 4……………………………………………………………………24

Figure 5……………………………………………………………………25

Figure 6……………………………………………………………………26

Figure 7……………………………………………………………………….....27

Figure 8……………………………………………………………………….....28

Figure 9……………………………………………………………………….....29

Supplementary Methods

mNGS analysis of BALF

Total RNA was extracted with a QIAamp® Viral RNA Kit (Qiagen) and ribosomal RNA was removed by a Ribo-Zero rRNA Removal Kit (Illumina). cDNA was made using reverse transcriptase and dNTPs (Thermo Fisher). Libraries were produced for the DNA and cDNA samples using a Nextera XT DNA Library Prep Kit (Illumina, San Diego, CA). Library pools were then loaded onto an Illumina Nextseq 550Dx sequencer for 75 cycles of single-end sequencing to generate approximately 20 million reads for each library.

Supplementary Results and Discussion

Genomic features

Phylogenetic analysis based on the whole-genome sequence similarities indicated that strain GZ2020^T belonged to *Nocardia* spp., and the closest known relatives were *Nocardia anaemiae* NBRC 100462^T, *Nocardia pseudovaccinii* NBRC 100343^T and *Nocardia vinacea* NBRC 16497^T. Illumina sequencing of GZ2020^T yielded 136,235,859 reads and 10.2 Gb, from which a complete genome of 9.39 Mb (GenBank accession number JAIRBR000000000) was assembled, with a G+C content of 66.89 mol%. The general gene characteristics of GZ2020^T are summarized in Supplementary Figure 6. By comparing the genome sequence of GZ2020^T with those of strains in the same branch (*Nocardia anaemiae* NBRC 100462^T, *Nocardia pseudovaccinii* NBRC 100343T and *Nocardia vinacea* NBRC 16497^T), ANI values <95% for identity alignment and dDDH values <70% were found (Supplementary Figure 7), which are below the thresholds defining a bacterial species (≥95–96% for ANI[1] and ≥70% for dDDH[2]). The ANI and dDDH results indicated that strain GZ2020^T represented a novel species of the genus *Nocardia*. Besides, further basic functional gene analysis also indicated that GZ2020^T was different from its relatives (Supplementary figure 8).

Physiological and chemical characteristics of GZ2020^T

Strain GZ2020^T grew well on CBA plates (Supplementary Figure 1). Under gross observation, colonies cultured on CBA plates were light yellow and circular and measured 0.5 to 1.0 mm in diameter after 3 to 5 days at 37 ℃. No diffusible pigment was produced on CBA. GZ2020^T was a gram-positive, weakly acid fast, strictly aerobic, nonmotile actinomycete. The strain grew at 20–45 ℃, pH 5.0–8.0 and NaCl concentrations of 0–2.0%, which is different from the results for the closest relatives (*Nocardia anaemiae* NBRC 100462^T, *Nocardia pseudovaccinii* NBRC 100343^T and *Nocardia vinacea* NBRC 16497^T). GZ2020^T was positive for catalase, oxidase and urease activities and nitrate reduction but negative for the degradation of gelatin, starch, casein, and Tween 20 and 80. H_2S was not produced. This pathogen was definitely susceptible to linezolid and possibly susceptible to moxifloxacin but resistant to amikacin, amoxicillin-clavulanic acid, ceftriaxone, clarithromycin, imipenem, minocycline, trimethoprim-sulfamethoxazole, and cefepime, while the relatives were susceptible to first-line antibiotic therapy, especially imipenem[3-6]. The physiological and biochemical features that distinguish this novel strain from closely related species are listed in Supplementary Table 4. The primary respiratory quinone was MK8 (H4, cyclic). The major fatty acids were C16:0 and 10-methyl C18:0. The major polar lipids were diphosphatidylglycerol, phosphatidylethanolamine, phosphatidylglycerol, unidentified lipids, unidentified phospholipids, unidentified aminolipids and phosphatidylinositol. The diagnostic diamino acid of peptidoglycan was *meso*-diaminopimelic acid. The basic whole-cell sugar pattern included galactose, arabinose and ribose.

Given the genotypic, phenotypic and chemotaxonomic characteristics of GZ2020^T differentiating it from the closest relatives, we conclude that GZ2020^T represents a novel species of the genus *Nocardia*. We propose establishing the new species *Nocardia* sp. nov. and designating strain GZ2020^T as the type strain.

Description of *Nocardia* GZ2020^T sp. nov.

Nocardia guangzhouensis (guang.zhou.en'sis.N.L. adj. *guangzhouensis*) belongs to The First Affiliated Hospital of Guangzhou Medical University, Guangzhou, Guangdong Province, PR China, where GZ2020^T was found.

Nocardia GZ2020^T is a gram-positive, weakly acid fast, aerobic, heterotrophic and nonmotile bacterium. Growth of this strain occurs at 20–45 ℃, pH 5.0–8.0 and NaCl concentrations of 0–2.0% (w/v). The strain is positive for catalase, oxidase and urease activities and nitrate reduction but negative for the degradation of cellulose, gelatin, starch, casein, and Tween 20 and 80. H_2S is not produced, and the strain could curdle but not peptonize milk. It utilizes sucrose, α-D-glucose, D-fructose, D-galactose, D-turanose, 3-methyl glucose, L-fucose, D-trehalose, sodium lactate and glycerol as carbon sources for energy production and cell growth but does not use D-maltose, D-cellobiose, gentiobiose, stachyose, D-raffinose, α-D-lactose, D-melibiose, D-mannose, D-fucose, or L-rhamnose. In addition, the strain can also use glycyl-L-proline, L-alanine, L-arginine, L-aspartic acid, and L-glutamic acid as nitrogen sources but cannot use N-acetyl neuraminic acid, inosine, D-aspartic acid, D-serine, L-serine, or L-pyroglutamic acid. The primary respiratory quinone is MK8 (H4, cyclic). The major fatty acids are C16:0 and 10-methyl C18:0. The major polar lipids are diphosphatidylglycerol, phosphatidylethanolamine, phosphatidylglycerol, unidentified lipids, unidentified phospholipids, unidentified aminolipids and phosphatidylinositol. The diagnostic diamino acid of peptidoglycan is *meso*-diaminopimelic acid. The basic whole-cell sugar pattern includes galactose, arabinose and ribose. The DNA G+C content of the type strain is 66.89 mol%. This pathogen is characterized by its low susceptibility to multiple antibiotics and is definitely susceptible to linezolid and possibly susceptible to moxifloxacin but resistant to conventional therapies, including amikacin, amoxicillin-clavulanic acid, ceftriaxone, clarithromycin, imipenem, minocycline, trimethoprim-sulfamethoxazole, and cefepime.

The type strain GZ2020^T (=GDMCC 4.187^T =JCM 34519^T) was isolated from the lung

tissue and BALF specimens of a patient at the First Affiliated Hospital of Guangzhou Medical University in Guangzhou, Guangdong, China.

Supplementary References

1. Richter M, Rosselló-Móra R. Shifting the genomic gold standard for the prokaryotic species definition. Proceedings of the National Academy of Sciences of the United States of America 2009;106:19126-31.
2. Chun J, Oren A, Ventosa A, et al. Proposed minimal standards for the use of genome data for the taxonomy of prokaryotes. International journal of systematic and evolutionary microbiology 2018;68:461-6.
3. Kim KK, Roth A, Andrees S, Lee ST, Kroppenstedt RM. *Nocardia* pseudovaccinii sp. nov. International journal of systematic and evolutionary microbiology 2002;52:1825-9.
4. Kinoshita N, Homma Y, Igarashi M, Ikeno S, Hori M, Hamada M. *Nocardia* vinacea sp. nov. Actinomycetologica 2001;15:1-5.
5. Kageyama A, Yazawa K, Nishimura K, Mikami Y. *Nocardia* anaemiae sp. nov. isolated from an immunocompromised patient and the first isolation report of *Nocardia* vinacea from humans. Nippon Ishinkin Gakkai Zasshi 2005;46:21-6.
6. *Nocardia* pseudovaccinii AR 368 is a mesophilic bacterium that was isolated from soil. (Accessed October 11, 2021, at https://bacdive.dsmz.de/strain/10763.)
7. Perçin D, Sümerkan B, Inci R. [Comparative evaluation of e-test and disk diffusion methods for susceptibility testing of *Nocardia* species]. Mikrobiyoloji bulteni 2011;45:274-9.
8. Hindler JA, Richter SS. Methods for Antimicrobial Dilution and Disk Susceptibility Testing of Infrequently Isolated Or Fastidious Bacteria: M45: Clinical and Laboratory Standards Institute; 2016.
9. Clinical Microbiology Unit CSoLM. Chinese expert consensus on metagenomics next-generation sequencing application on pathogen detection of infectious diseases. Chinese Journal of Laboratory Medicine 2021;44:107-20.

Supplementary Table 1. Genomes and associated accession numbers in the phylogenetic tree

Genome	Associated accession number
Nocardia abscessus NBRC 100374^T	BAFP00000000
Nocardia acidivorans NBRC 108247^T	BDAW00000000
Nocardia africana NBRC 100379^T	BDAV00000000
Nocardia alba DSM 44684^T	SMFR00000000
Nocardia altamirensis NBRC 108246^T	BDAY00000000
Nocardia amamiensis NBRC 102102^T	BDBA00000000
Nocardia amikacinitolerans NBRC 108937^T	BDAU00000000
Nocardia anaemiae NBRC 100462^T	BDAZ00000000
Nocardia aobensis NBRC 100429^T	BAFQ00000000
Nocardia araoensis NBRC 100135^T	BAFR00000000
Nocardia arizonensis NBRC 108935^T	BDCT00000000
Nocardia arthritidis NBRC 100137^T	BDBB00000000
Nocardia asiatica NBRC 100129^T	BAFS00000000
Nocardia asteroides NCTC 11293^T	LR134352
Nocardia beijingensis NBRC 16342^T	BDBC00000000
Nocardia bhagyanarayanae DSM 103495^T	VFPG00000000
Nocardia brasiliensis NCTC 11294^T	UGSN00000000
Nocardia brevicatena NBRC 12119^T	BAFU00000000
Nocardia caishijiensis DSM 44831^T	VMSD00000000
Nocardia carnea NBRC 14403^T	BAFV00000000
Nocardia cerradoensis NBRC 101014^T	BAFW00000000
Nocardia colli CICC 11023^T	VXLC00000000
Nocardia concava NBRC 100430^T	BAFX00000000
Nocardia crassostreae NBRC 100342^T	BDCH00000000
Nocardia cyriacigeorgica DSM 44484^T	VBUR00000000
Nocardia donostiensis X1654^T	MUKP00000000
Nocardia exalbida NBRC 100660^T	BAFZ00000000
Nocardia farcinica NCTC 11134^T	LN868938
Nocardia flavorosea NBRC 108225^T	BDCG00000000
Nocardia fluminea DSM 44489^T	PJMW00000000
Nocardia grenadensis NBRC 108939^T	BDCJ00000000
Nocardia harenae NBRC 108248^T	BDBH00000000
Nocardia higoensis NBRC 100133^T	BAGA00000000
Nocardia ignorata DSM 44496^T	SNXK00000000
Nocardia inohanensis NBRC 100128^T	BDBK00000000
Nocardia jejuensis NBRC 103114^T	BDBU00000000
Nocardia jiangxiensis NBRC 101359^T	BAGB00000000
Nocardia jinanensis NBRC 108249^T	BDBO00000000
Nocardia kruczakiae NBRC 101016^T	BDBL00000000
Nocardia lijiangensis NBRC 108240^T	BDBP00000000
Nocardia mangyaensis Y48^T	CP018082

Nocardia mexicana DSM 44952^{T}	QQAZ00000000
Nocardia mikamii NBRC 108933^{T}	BDCM00000000
Nocardia miyunensis NBRC 108239^{T}	BDBQ00000000
Nocardia neocaledoniensis DSM 44717^{T}	QGTL00000000
Nocardia niigatensis NBRC 100131^{T}	BAGC00000000
Nocardia ninae NBRC 108245^{T}	BJXA00000000
Nocardia niwae NBRC 108934^{T}	BDCK00000000
Nocardia nova NBRC 15556^{T}	BDBN00000000
Nocardia otitidiscaviarum NCTC 1934^{T}	UGRY00000000
Nocardia paucivorans NBRC 100373^{T}	BAGE00000000
Nocardia pneumoniae NBRC 100136^{T}	BAGF00000000
Nocardia pseudobrasiliensis DSM 44290^{T}	QQBC00000000
Nocardia pseudovaccinii NBRC 100343^{T}	BDBY00000000
Nocardia puris DSM 44599^{T}	QNRE00000000
Nocardia rhamnosiphila NRRL B-24637^{T}	JOAJ00000000
Nocardia salmonicida subsp. salmonicida NBRC 13393^{T}	BDBR00000000
Nocardia seriolae NBRC 15557^{T}	BJWY00000000
Nocardia shimofusensis NBRC 100134^{T}	BDBT00000000
Nocardia sienata NBRC 100364^{T}	BDBX00000000
Nocardia guangzhouensis JCM 34519^{T}	JAIRBR000000000
Nocardia speluncae NBRC 108251^{T}	BDBZ00000000
Nocardia stercoris NEAU-LL90^{T}	RFFH00000000
Nocardia takedensis NBRC 100417^{T}	BAGG00000000
Nocardia tenerifensis DSM 44704^{T}	QJKF00000000
Nocardia testacea NBRC 100365^{T}	BAGJ00000000
Nocardia thailandica NBRC 100428^{T}	BAGK00000000
Nocardia transvalensis NBRC 15921^{T}	BAGL00000000
Nocardia uniformis NBRC 13702^{T}	BDCE00000000
Nocardia vaccinii NBRC 15922^{T}	BDCC00000000
Nocardia vermiculata NBRC 100427^{T}	BDCA00000000
Nocardia veterana NBRC 100344^{T}	BAGM00000000
Nocardia vinacea NBRC 16497^{T}	BAGN00000000
Nocardia vulneris NBRC 108936^{T}	BDCI00000000
Nocardia xishanensis NBRC 101358^{T}	BDCF00000000
Nocardia yamanashiensis NBRC 100130^{T}	BDCD00000000
Nocardia yunnanensis CFHS0054^{T}	CP032568
Rhodococcus rhodochrous NCTC 10210^{T}	LT906450

The strains highlighted in yellow are GZ2020^{T} and its relatives.

Supplementary Table 2. Physiological and chemical characteristics of GZ2020^{T}

Characteristics	Result
**Chemotaxonomic characteristics**	
Major menaquinone	MK8 (H4, cyclic)
Major fatty acids	C16: 0 and 10-methyl C18: 0
Characteristic whole-cell sugars	Galactose, arabinose and ribose
Wall DAP	*Meso*-diaminopimelic acid
Mycolic acids	+
Main polar lipids	DPG and PE
**Routine physiological and biochemical tests**	
Temperature	20–45℃
pH	5–8
NaCl	0–2%
Tween20	-
Tween80	-
Hydrolyzed starch	-
Hydrolyzed casein	-
Anaerobic culture	-
Nitrate reduction	+
Gelatin	-
Urease	+
H_2S	-
Milk coagulation and peptonization	Solidified but not peptonized
H_2O_2	+
Oxidase	-

DAP: diaminopimelic acid; DPG: diphosphatidylglycerol; PE: phosphatidylethanolamine.

**Supplementary Table 3. Utilization by GZ2020^{T}**					
Negative Control	Dextrin	D-Maltose	D-Trehalose	D-Cellobiose	Gentiobiose
-	-	-	W	-	-
D-Raffinose	α-D-Lactose	D-Melibiose	β-Methyl-D-Glucoside	D-Salicin	N-Acetyl-D-Glucosamine
-	-	-	W	W	-
α-D-Glucose	D-Mannose	D-Fructose	D-Galactose	3-Methyl Glucose	D-Fucose
W	-	W	W	W	-
D-Sorbitol	D-Mannitol	D-Arabitol	myo-Inositol	Glycerol	D-Glucose-
-	W	W	W	+	W
Gelatin	Glycyl-L-Proline	L-Alanine	L-Arginine	L-Aspartic Acid	L-Glutamic Acid
-	+	W	W	W	+
Pectin	D-Galacturonic Acid	L-Galactonic Acid Lactone	D-Gluconic Acid	D-Glucuronic Acid	Glucuronamide
-	W	-	W	W	W
p-Hydroxy-Phenylacetic Acid	Methyl Pyruvate	D-Lactic Acid Methyl Ester	L-Lactic Acid	Citric Acid	α-Keto-Glutaric Acid
-	W	W	-	+	+
Tween 40	γ-Amino-Butyric Acid	α-Hydroxy-Butyric Acid	β-Hydroxy-D,L-Butyric Acid	α-Keto-Butyric Acid	Acetoacetic Acid
+	W	W	+	-	-
Sucrose	D-Turanose	Stachyose	Positive Control	pH 6	pH 5
W	W	-	+	W	-
N-Acetyl-β-D-Mannosamine	N-Acetyl-D-Galactosamine	N-Acetyl Neuraminic Acid	1% NaCl	4% NaCl	8% NaCl

W	W	-	W	-	-
L-Fucose	L-Rhamnose	Inosine	1% Sodium	Fusidic Acid	D-Serine
W	-	-	W	-	-
D-Fructose-6-PO4	D-Aspartic Acid	D-Serine	Troleandomycin	Rifamycin SV	Minocycline
-	-	-	-	+	W
L-Histidine	L-Pyroglutamic	L-Serine	Lincomycin	Guanidine HCl	Niaproof 4
+	W	-	-	-	-
Mucic Acid	Quinic Acid	D-Saccharic Acid	Vancomycin	Tetrazolium Violet	Tetrazolium Blue
W	W	W	W	W	-
D-Malic Acid	L-Malic Acid	Bromo-Succinic Acid	Nalidixic Acid	Lithium Chloride	Potassium Tellurite
W	W	W	-	-	W
Propionic Acid	Acetic Acid	Formic Acid	Aztreonam	Sodium Butyrate	Sodium Bromate
-	W	-	+	-	-

"-": negative; "+": positive; "W": weakly positive

Supplementary Table 4. Comparisons between GZ2020^T and other species

	GZ2020^T	*Nocardia anaemiae*[a]	*Nocardia vinacea*[b]	*Nocardia pseudovaccinii*[c]
**Chemotaxonomic characteristics**				
Major menaquinone	MK8 (H4, cyclic)	MK8 (H4, cyclic)	MK8(H4, cyclic)	MK8 (H4, cyclic)
Major fatty acids	C16: 0, 10-methyl C18: 0	ND	ND	C16: 0, 10-methyl C18: 0
Characteristic whole-cell sugars	galactose, arabinose and ribose	galactose and arabinose	galactose and arabinose	galactose and arabinose
Wall DAP	meso-diaminopimelic acid	meso-diaminopimelic acid	meso-diaminopimelic acid	meso-diaminopimelic acid
Mycolic acids	+	+	+	+
**Utilization of**				
D-galactose	w	+[d]	+[d]	-
L-rhamnose	-	-[e]	-[e]	-
Sucrose	+	ND	-	-
D-turanose	W	ND	ND	-
D-arabitol	W	ND	ND	+
myo-Inositol	w	-[f]	-	+[g]
N-acetyl D-glucosamine	-	ND	ND	+
L-alanine	W	ND	ND	+
L-serine	-	ND	ND	-
α-D-glucose	w	+[h]	+[h]	ND
D-maltose	-	-[i]	-[i]	ND
D-mannose	-	-[j]	-[j]	ND
D-sorbitol	-	+[k]	+	ND
D-fructose	w	ND	+	ND
D-raffinose	-	ND	-[l]	ND
**Decomposition of**				

Starch	-	ND	-	ND
Casein	-	-	-	ND
**Biochemical tests**				
Nitrate reductase	+	ND	+	ND
Urea hydrolysis	+	+	+	ND
**Susceptibility test**				
Imipenem	Resistant	Susceptible	Susceptible	ND
Tobramycin	Resistant[m]	Susceptible	Susceptible	ND
**Growth at**				
37 ℃	+	+	+	+
45 ℃	+	-	-	-
4% NaCl	-	ND	+	ND

"-": negative; "+": positive; "W": weakly positive; ND: No data. a: Data from Kageyama et al[5]. b: Data from Kinoshita [4] et al and Kageyama et al[5]. c: Data from Kim et al[3] and BacDive[6]. d: Data were reported as galactose in the reference. e: Data were reported as rhamnose in the reference. f: Data were reported as –i-inositol in the reference. g: Data were reported as inositol in the reference. h: Data were reported as glucose in the reference. i: Data were reported as maltose in the reference. j: Data were reported as mannose in the reference. k: Data were reported as sorbitol in the reference. l: Data were reported as raffinose in the reference. m: The result was interpreted according to the CLSI breakpoints of disc diffusion tests for Staphylococcus spp. just as other researches[7,8].

**Supplementary Table 5. Laboratory testing**							
**Measure**	**Reference range**	**Hospital day 1**	**Hospital day 4**	**Hospital day 6**	**Hospital day 7**	**Hospital day 12**	**Hospital days 39 to 42**
**Routine blood examination**							
White blood cell count, × 10^9/L	4.0–10.0	17	12.3	8.7	14.6	11	9.7
Neutrophil count, × 10^9/L	1.80–8.00	14.6	10.3	7.3	12.6	9.4	7.8
Neutrophil ratio, %	40–70	85.9	84	83.8	86.3	85.5	79.7
Lymphocyte count, × 10^9/L	0.90–5.20	1.5	0.9	0.6	0.7	0.8	1.4
Hemoglobin, g/L	110–150	118	91	85	89	88	58
Platelet count, × 10^9/L	100–400	518	317	278	333	436	193
**Inflammatory marker**							
PCT, ng/ml	0-0.05	0.2	/	/	/	/	<0.05
ESR, mm/h	0-20	64	/	/	/	/	16
**Coagulation function**							
PT, S	11–14.50	14	13.5	13	13.8	12.9	12.8
FIB, g/L	2–4	4.37	4	3.68	4.49	4.89	2.37
APTT, S	28–42.80	60.7	46.9	39.7	40.9	43.2	38.4
D-dimer, ng/mL FEU	68–494	546	563	481	467	369	184
**Liver function analysis**							
ALT, U/L	5–40	30	/	/	/	9.5	15.7
AST, U/L	5–40	42.1	/	/	/	19.5	18.8
TP, g/L	65–85	65.8	/	/	/	52.9	59.5
TBIL, µmol/L	1.7–22.20	21.3	/	/	/	7.8	10.7
**Enzymatic indicators**							
CK, U/L	10–190	52	/	81.5	19.5	9.5	25.1
CKMB, U/L	3–25	8	/	9	4	6	5
LDH, U/L	109–255	208.8	/	172.1	182.5	166.2	110

aTnI, μg/L	0–0.04	0	/	0	0	/	0
MYO, μg/L	<70	20.9	/	48.7	16.9	/	8.6
**Biochemical function analysis**							
BUN, mmol/L	2.9–7.2	9	2.6	/	/	1.1	2.5
Cr, μmol/L	44–133	58.9	57.6	/	/	44	52.9
K, mmol/L	3.5–5.3	4.04	2.9	2.37	3.88	3.69	3.13
Na, mmol/L	134–145	127	125.1	111.7	127.5	126.8	141
Cl, mmol/L	96–111	89.4	82.3	74.3	91	89.9	103.2
Ca, mmol/L	2.03–2.7	2.35	2.07	1.93	2.03	1.96	2.23

"/" indicates that the value was not detected at the time. PCT: procalcitonin; ESR: erythrocyte sedimentation rate; PT: prothrombin time; FIB: fibrinogen; APTT: activated partial thromboplastin time; S: second; ALT: alanine transaminase; AST: aspartate aminotransferase; TP: total protein; TBIL: total bilirubin; CK: creatine kinase; CKMB: creatine kinase MB; LDH: lactate dehydrogenase; aTnI: cardiac troponin I; MYO: myoglobin.

Supplementary Table 6. Detection of respiratory pathogens				
**Measure**	Date*	Hospital day	Samples	Results
**Viruses**				
IgM antibody tests[a]	July 8	2	Blood serum	Negative
Cytomegalovirus nucleic acid testing	July 20	14	BALF	Negative
**Tuberculosis**				
Smear test	July 8	2	Sputum	Negative
Tuberculosis nucleic acid test (TB-DNA)	July 10	4	Sputum	Negative
Smear test	July 10	4	Sputum	Negative
Smear test	July 11	5	Sputum	Negative
Smear test	July 14	8	Sputum	Negative
Tuberculosis nucleic acid test (TB-DNA)	July 18	12	Sputum	Negative
T-cell assay for tuberculosis infection (immunospot assay)	July 9	3	Blood serum	Negative
Smear test	July 18	12	Bronchoscopy brushing specimens	Negative
Smear test	July 18	12	Bronchoscopy brushing specimens	Negative
**Fungi**				
Fungal culture	July 13	7	Venous blood	Negative
Fungal culture	July 14	8	Venous blood	Negative
Fungal culture	July 18	12	Venous blood	Negative
G test	July 8	2	Blood serum	Negative
Fungal antigen test	July 9	3	Blood serum	Negative
Total IgE determination against Aspergillus	July 10	4	Blood serum	Negative

Fungus culture	July 23	17	Lung tissue	Negative
Fungal culture	July 24	18	Lung tissue	Negative
Fungal culture	July 15	9	Sputum	Negative
Fungal culture	July 18	12	Sputum	Negative
**Bacteria**				
Smear test	July 8	2	Sputum	Negative
Bacterial culture	July 10	4	Sputum	Negative
Smear test	July 11	5	Sputum	Negative
Bacterial culture	July 13	7	Sputum	Negative
Smear test	July 18	12	Sputum (from bronchoscopy)	Negative
Smear test	July 20	14	Sputum	Negative
Bacterial culture	July 13	7	Venous blood	Negative
Bacterial culture	July 14	8	Venous blood	Negative
Bacterial culture	July 18	12	Venous blood	Negative
Bacterial culture	July 24	18	Lung tissue	*Nocardia* (susceptible to linezolid)
Bacterial culture	July 24	18	BALF	*Nocardia* (susceptible to linezolid)
**mNGS[b]**				
Viruses	July 20	14	BALF	Negative
Tuberculosis	July 20	14	BALF	Negative
Fungi	July 20	14	BALF	Negative
Bacteria	July 20	14	BALF	*Nocardia* (nontypable)

*: The time when the test results were available. a: Including detection of respiratory syncytial virus, respiratory adenovirus, influenza A and B viruses and parainfluenza virus. b: More details were shown in Table S8. mNGS: metagenomic next-generation sequencing.

**Supplementary Table 7.** mNGS analysis of the patient’s BALF*	
**Genus**	**Number of sequences**
**Bacteria***	
Nocardia	49850
Mycobacterium	337
Sphingomonas	75
Rhodococcus	71
Streptomyces	43
Amycolatopsis	35
Gordonia	32
Corynebacterium	24
Mycoplasma	23
Pseudomonas	23
Actinomadura	22
Propionibacterium	21
Acinetobacter	20
Burkholderia	18
Actinoplanes	16
Moraxella	13
Allokutzneria	12
Lautropia	11
Delftia	11
Methylobacterium	9
Kibdelosporangium	9
Streptosporangium	7
Tsukamurella	6
Cryptosporangium	6
Saccharothrix	6
Actinospica	6
Phenylobacterium	5
Microlunatus	5
Crossiella	5
Enterococcus	4
Microbacterium	4
Arthrobacter	4
Sphingobium	4
Millisia	4
Actinoalloteichus	4
Micromonospora	4
Actinokineospora	4
Catenulispora	4
Staphylococcus	3
Micrococcus	3
Actinomyces	3

Bacillus	3
Nakamurella	3
Microbispora	3
Dactylosporangium	3
**Fungi**	
Candida	30
Fonsecaea	19
**Special pathogens in mNGS#**	
Nocardia(non-typable)	49850
Mycobacterium_tuberculosis_complex	0
Aspergillus	0
Cryptococcus	0
Mucor	0
Talaromyces marneffei	0
**Virus**	/
**parasite**	/
**Total**	50876

mNGS: metagenomic next-generation sequencing. BALF: bronchiolar lavage fluid.

*: Only sequences greater than 3 were shown. #:According to expert consensus[9]

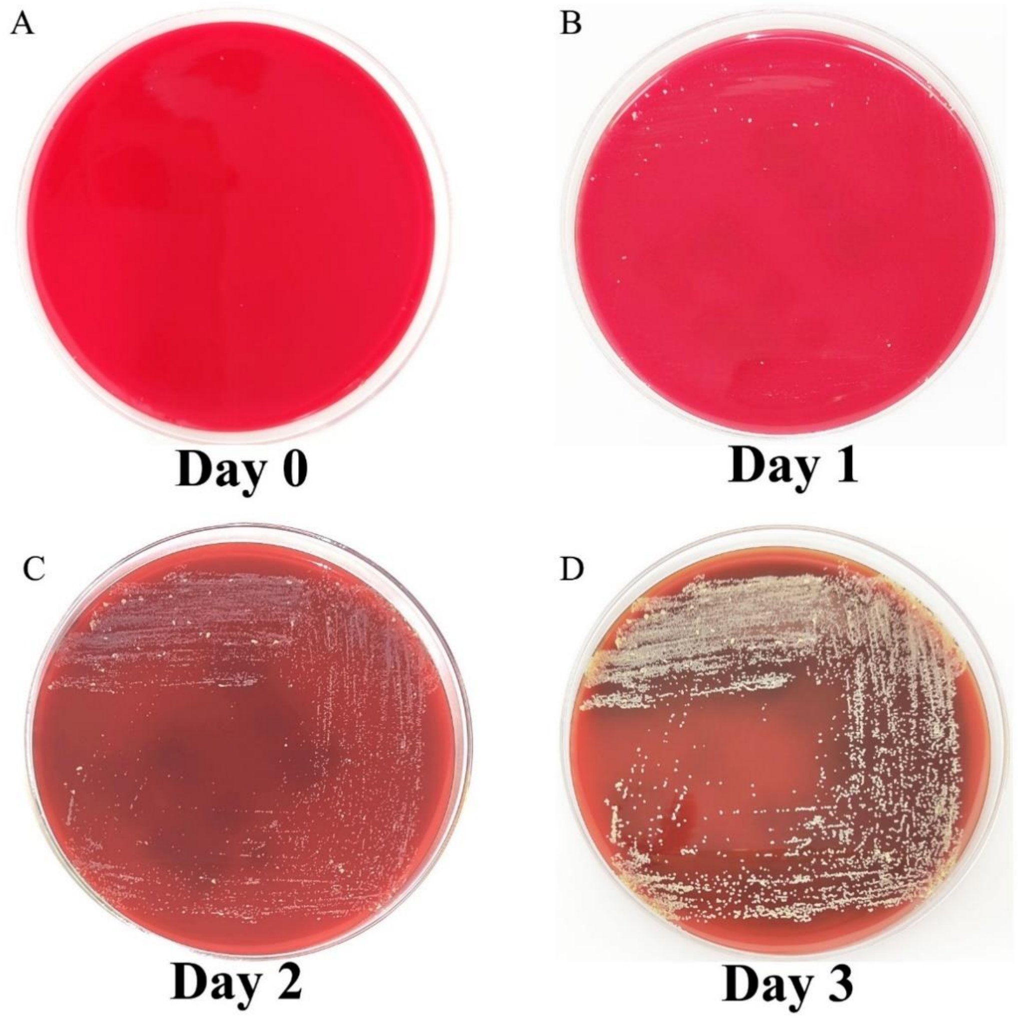

Supplementary Figure 1. Growth rate and gross appearance of GZ2020^T

A, B, C, and D represent the growth status of GZ2020^T at different time points. On day 0, the bacterial colonies were picked with a sterilized inoculation loop and streaked onto the surface of nutrient agar by the quadrant streak plate method to obtain separate colonies. By gross appearance observation, GZ2020^T seemed dry, small and rough.

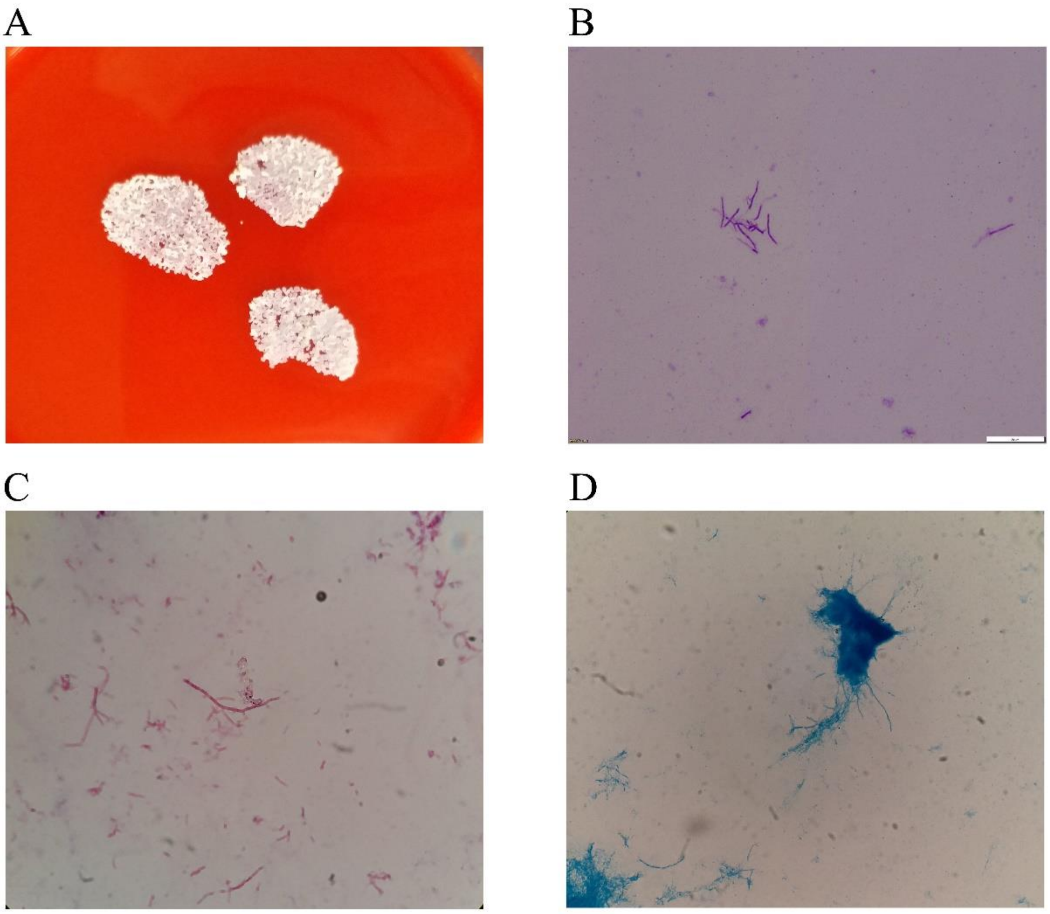

Supplementary Figure 2. Staining and microscopic examination of GZ2020^{T}.

A: Gross features of bacteria (GZ2020^{T}) isolated from the patient's alveolar lavage fluid (cultured in CBA for 3-5 days). Gram staining (B), weak acid-fast staining (C), and acid-fast staining (D) were used, and under an optical microscope (400X magnification), GZ2020^{T} was observed to be beaded and rod-shaped, with branching filaments. Bacteriologic analysis revealed positive Gram staining and weak acid-fast staining but negative acid-fast staining.

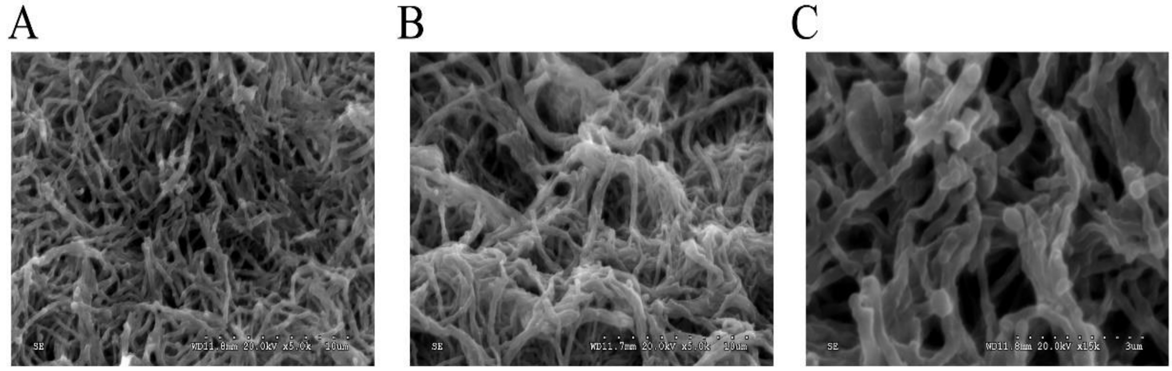

Supplementary Figure 3. Scanning electron microscopy analysis of GZ2020^{T}.

A, B (5000X magnification) and C (15000X magnification) reveal the characteristics of the bacterial surface, showing a filamentous network covered with an opaque biofilm matrix.

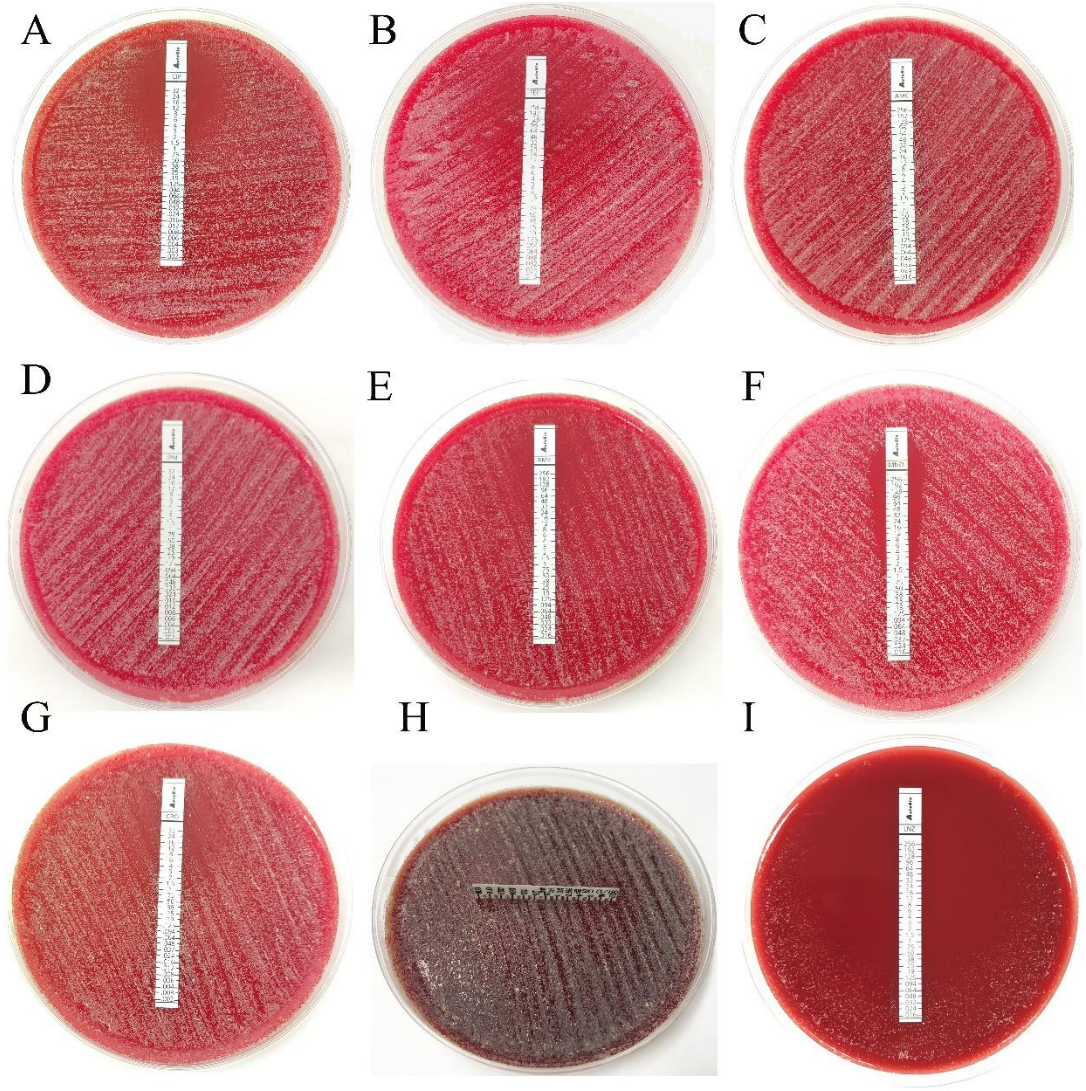

Supplementary Figure 4. Drug susceptibility tests (minimum inhibitory concentration assays).

CIP: ciprofloxacin (A); FEP: cefepime (B); AMC: amoxicillin and clavulanate potassium (C); IPM: imipenem (D); AMK: amikacin (E); MNO: minocycline (F); CRO: ceftriaxone (G); SXT: trimethoprim-sulfamethoxazole (H); LNZ: linezolid (I). Interpretations of these results are shown in Fig. 2A. The above results indicated that GZ2020^{T} was susceptible to only linezolid.

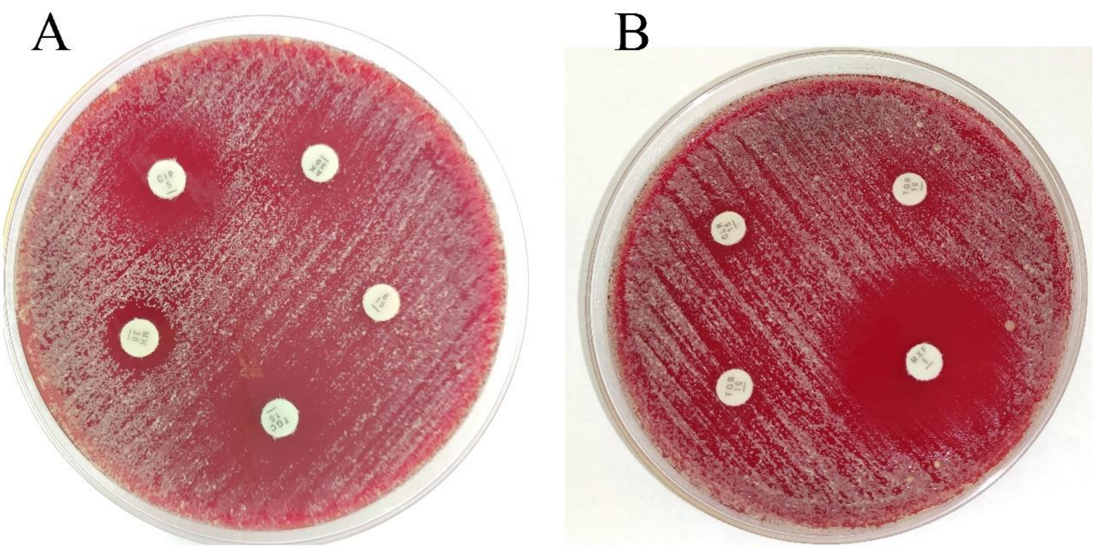

Supplementary Figure 5. Drug susceptibility tests (disc diffusion tests).

CIP: ciprofloxacin, MH: minocycline, TGC: tigecycline; E: erythromycin; AK: amikacin; TOB: tobramycin; CLR: clarithromycin; MXF: moxifloxacin. The diameters of the bacteriostasis circles of erythromycin and tigecycline were 6 mm and 13 mm, respectively; the interpretations of other results are shown in Fig. 2A. These results were used as a complement when the E-test was not available; therefore, only the results for tobramycin, clarithromycin and moxifloxacin were interpreted according to disc diffusion tests, showing that GZ2020^{T} was resistant to tobramycin and clarithromycin and possibly susceptible to moxifloxacin.

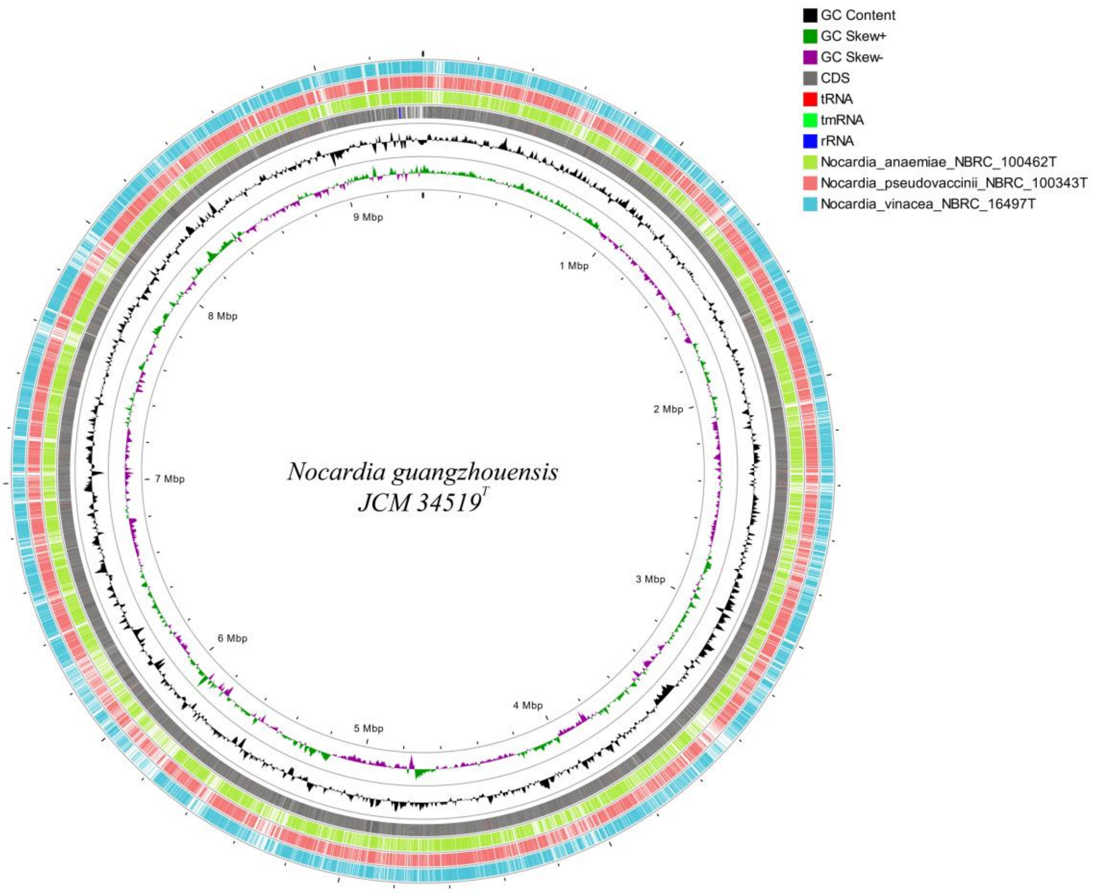

Supplementary Figure 6. **Genomic features of GZ2020^T**

Circular genomes map comparing the sequence of GZ2020^T to other *Nocardia* genomes using proksee that base on CGView.js engine（https://proksee.ca/）. Starting from the innermost ring the feature rings depict: 1. Genome length; 2. GCskew; 3. GC content; 4. Annotated gene feature with Prokka. The next 3 rings show regions of sequence similarity detected by BLAST comparisons conducted between CDS translations from the genome of GZ2020^T and its 3 relatives.

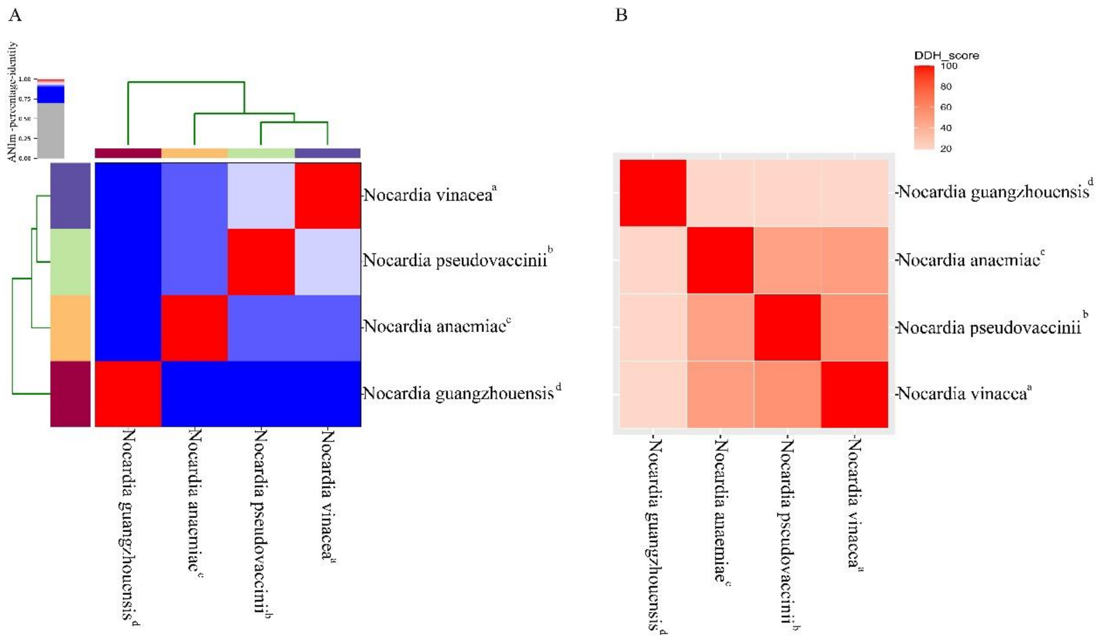

Supplementary Figure 7. ANI and dDDH analysis between GZ2020^T and *Nocardia anaemiae*, *Nocardia pseudovaccinii* and *Nocardia vinacea*.

ANI: average nucleotide identity; dDDH: digital DNA:DNA hybridization.

A: ANI analysis of percent identity. B: dDDH analysis of GZ2020^T and other relatives.

a: *Nocardia vinacea* NBRC 16497^T.

b: *Nocardia pseudovaccinii* NBRC 100343^T; c: *Nocardia anaemiae* NBRC 100462^T; d: *Nocardia guangzhouensis* JCM 34519^T. A shows ANI values <95%, and B shows dDDH values <70% in the alignment of GZ2020^T with the other 3 pathogens, indicating that GZ2020^T is different from these pathogens and represents a novel *Nocardia* species.

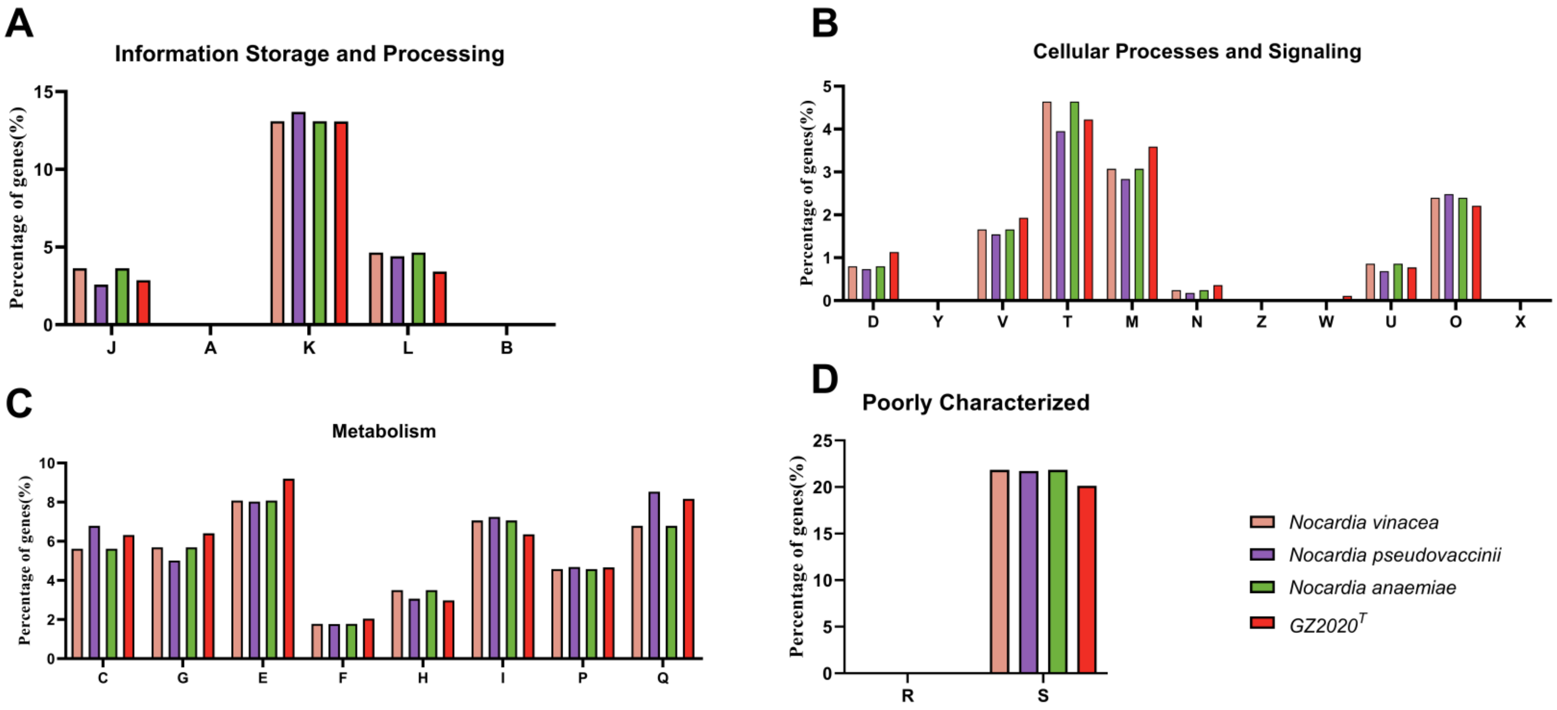

Supplementary Figure 8. Basic functional gene analysis of GZ2020^{T}

The gene analysis comprises four aspects: information storage and processing, cellular processes and signaling, metabolism and some other poorly characterized areas. The abscissa represents different genes while the ordinate represents the proportions of the number of the gene among all basic functional genes.

J: Translation, ribosomal structure and biogenesis. A: RNA processing and modification. K: Transcription. L: Replication, recombination and repair. B: Chromatin structure and dynamics.

D: Cell cycle control, cell division, chromosome partitioning. Y: Nuclear structure. V: Defense mechanisms. T: Signal transduction mechanisms. M: Cell wall/membrane/envelope biogenesis. N: Cell motility. Z: Cytoskeleton. W: Extracellular structures. U: Intracellular trafficking, secretion, and vesicular transport. O: Posttranslational modification, protein turnover, chaperones. X: Mobilome: prophages, transposons.

C: Energy production and conversion. G: Carbohydrate transport and metabolism. E: Amino acid transport and metabolism. F: Nucleotide transport and metabolism. H: Coenzyme transport and metabolism. I: Lipid transport and metabolism. P: Inorganic ion transport and metabolism. Q: Secondary metabolites biosynthesis, transport and catabolism. R: General function prediction only. S: Function unknown.

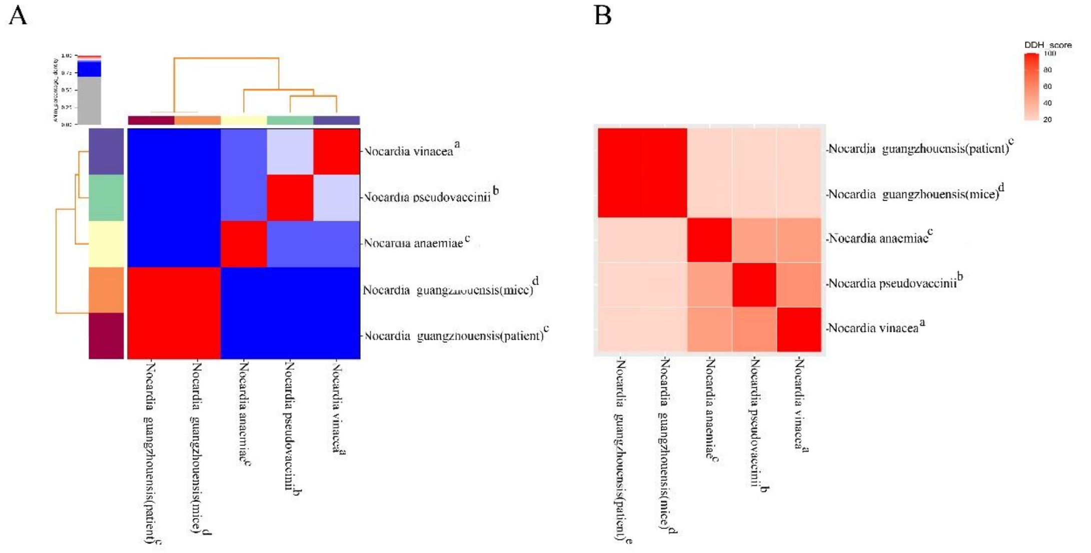

Supplementary Figure 9. ANI and dDDH analysis between GZ2020^T and *Nocardia* species isolated from the in vivo experiments.

ANI: average nucleotide identity; dDDH: digital DNA:DNA hybridization.

a: *Nocardia vinacea* NBRC 16497^T.b: *Nocardia pseudovaccinii* NBRC 100343^T; c: *Nocardia anaemiae* NBRC 100462^T; d: *Nocardia guangzhouensis* JCM 34519^T isolate from the patient. e: *Nocardia guangzhouensis* JCM 34519^T isolated from the mice.

A: ANI analysis of percent identity. B: dDDH analysis of GZ2020^T and other relatives.

Both A and B showed ANI and dDDH values > 99% from the alignment of GZ2020^T and *Nocardia* species isolated from mice, which indicated that the 2 isolates are the same species and do not differ.
